# Role of guaifenesin in the management of chronic bronchitis and upper respiratory tract infections

**DOI:** 10.1186/s40248-017-0113-4

**Published:** 2017-12-11

**Authors:** Helmut H. Albrecht, Peter V. Dicpinigaitis, Eric P. Guenin

**Affiliations:** 1 Florida International University, Herbert Wertheim College of Medicine, 11200 SW 8th St., GL 495, Miami, FL 33199 USA; 2 Albert Einstein College of Medicine and Montefiore Medical Center, 1825 Eastchester Road, Bronx, NY 10461 USA; 3 Reckitt Benckiser, LLC, 399 Interpace Parkway, Parsippany, NJ 07054 USA

**Keywords:** Guaifenesin, Mucus, Cough, Expectorant, Chronic bronchitis, Respiratory tract infections, Mucociliary clearance; over-the-counter (OTC), Extended-release (ER) formulation, Mucoactive agents

## Abstract

Guaifenesin, a mucoactive drug, acts by loosening mucus in the airways and making coughs more productive. It is used for relief of wet cough and chest congestion due to the common cold, and remains the only legally marketed expectorant in the US (per OTC Monograph). An ingredient in numerous over-the-counter (OTC) cough/cold medications, guaifenesin has a secondary indication for use in stable chronic bronchitis (professional indication). Clinical pharmacology and patient studies support the clinical utility of guaifenesin in respiratory conditions where mucus hypersecretion is prevalent: acute upper respiratory tract infections (URTIs), stable chronic bronchitis, and possibly rhinosinusitis. Guaifenesin has a well-established and favorable safety and tolerability profile in adult and pediatric populations. Its dosing range (200–400 mg 4-hourly, up to 6× daily) allows flexible dose titration to allow an increase of plasma concentrations. Multiple daily doses are needed to maintain 24-h therapeutic effect with immediate-release formulations. Extended-release guaifenesin tablet formulations are available, providing convenience with 12-hourly dosing and portability compared to liquids. Guaifenesin is considered as a safe and effective expectorant for the treatment of mucus-related symptoms in acute URTIs and stable chronic bronchitis. Its clinical efficacy has been demonstrated most widely in chronic respiratory conditions, where excess mucus production and cough are more stable symptoms. Progress is being made to establish clinical models and measures that are more appropriate for studying symptomatic relief with guaifenesin in acute respiratory infections. This will help generate the up-to-date and high-quality data needed to optimize guaifenesin’s effectiveness in established uses, and in new respiratory indications associated with mucus hypersecretion.

## Background

Respiratory conditions have been known throughout most of recorded medical history, and today mortality and morbidity associated with respiratory conditions represent a substantial global health burden. Statistics show over a hundred million people living with chronic respiratory conditions worldwide [1], while acute respiratory infections are among the most common reasons for physician office visits [2].

Pathological hypersecretion of mucus is a common feature in many acute and chronic respiratory conditions. Expectorants are used empirically to treat cough with an underlying cause of pathological mucus, by targeting various mechanisms that promote increased mucus hydration and clearance from the respiratory tract. Guaifenesin, or glyceryl guaiacolate ether (GGE), is an oral expectorant and a common ingredient in prescription and over-the-counter (OTC) medicines for respiratory conditions. Despite its wide use for the symptomatic management of chest congestion and cough associated with acute upper respiratory tract infections (URTIs), such as the common cold, guaifenesin’s precise mechanism of action has not been fully elucidated.

The use of guaifenesin as a natural remedy dates back to the 1500s, when guaiac tree extracts were used by Native Americans to treat various illnesses (Table 1). The drug was first accepted in 1952 by the US Food and Drug Administration (FDA); in 1989, it was included in the Final Monograph for ‘Cold, Cough, Allergy, Bronchodilator, and Anti-asthmatic Drug Products for Over-the-Counter Human Use’ [3], 21 CFR 341. Inclusion in the Monograph established guaifenesin as a safe and effective expectorant for the symptomatic treatment of acute URTIs and also allowed use of the drug in stable chronic bronchitis. Today, guaifenesin is still the only OTC expectorant legally marketed in the US.

**Table 1:** Brief history of guaifenesin and its regulatory path in the US

Time	Key events
Pre-1500s	Used as natural remedy by Native Americans
1500s	Guaiac extract used as stimulant remedies, e.g. for sore throat, syphilis
1800s	Guaiac extract used to treat respiratory diseases in Europe
1952	First accepted by US Food and Drug Administration (FDA)
1989	Guaifenesin was reclassified to Category I (generally recognized as safe and effective) and was included in the Final Monograph (Cold, Cough, Allergy, Bronchodilator, and Antiasthmatic Drug Products for Over-the-Counter Human Use, 21 CFR 341) as an expectorant for the symptomatic treatment of colds and stable chronic bronchitis.
2002	12-h extended-release (ER) guaifenesin bi-layer tablets were approved by the FDA. From 2007, the FDA removed all marketed, but unapproved, timed-release guaifenesin products from the market.

The purpose of this article is to review scientific evidence for the use of guaifenesin in different respiratory conditions and to summarize the key clinical studies. As a single-ingredient product, guaifenesin has an acceptable safety profile in both adult and pediatric populations. We describe recent advances in the understanding of guaifenesin’s mechanism of action and briefly discuss the rationale for its use in the context of its pharmacology, pharmacodymanics, and clinical efficacy profile.

### Mucus in airway function and disease

The respiratory tract is covered with a layer of mucus, which maintains airway humidification and acts as a protective barrier to inhaled particles and microorganisms. Mucus entraps inhaled particles and is then transported out of the lungs by the sweeping movements of epithelial cilia—a process termed mucociliary clearance (MCC) [4]—before being swallowed or expectorated. A dynamic balance of production, secretion, and clearance of mucus is needed to maintain airway function and health.

Respiratory conditions can dramatically alter airway mucus composition and properties. Upregulation of mucins, high molecular-weight extracellular mucopolysaccharides that are critical components of mucus, increases mucus viscosity; this can worsen congestion [5]. Pathological overproduction and hypersecretion of mucus feature prominently in chronic respiratory conditions such as chronic bronchitis, chronic obstructive pulmonary disease (COPD) and asthma [4, 6]. In fact, mucus hypersecretion has been described as a hallmark of the chronic bronchitis “phenotype” [7]. Since excessive respiratory mucus dramatically hinders MCC and serves as a trigger for cough [4], normalization of pathological mucus is a central goal of many therapeutic interventions in respiratory disease.

### Guaifenesin: multiple effects on pathological mucus

Therapy with mucoactive drugs is an important factor in the treatment of respiratory conditions in which mucus hypersecretion is prevalent. A large number of drugs acting directly or indirectly on mucus have been well studied and reviewed [5, 8–12].

There are four main classes of mucoactive drugs with different mechanisms of action (Table 2). Out of these, only mucolytic and expectorant drugs act directly on mucus properties or its secretion. Earlier studies showed that guaifenesin has multiple effects on mucus, such as increasing the volume of bronchial secretions and decreasing mucus viscosity. This modulation of airway secretions enhances their clearance by promoting more effective expectoration. Guaifenesin may also have direct effects on respiratory tract epithelial cells, including suppressed mucin production, reduced mucus viscoelasticity, and improved MCC [13]. One study indicated that guaifenesin does not act directly on mucus viscosity [14]. The effects of guaifenesin are not limited to affecting mucus consistency (e.g., increasing mucus hydration or altering viscoelasticity); it appears that the drug directly or indirectly targets multiple processes, including the inhibition of cough reflex sensitivity [15, 16].

**Table 2:** Main classes of mucoactive drugs

Mucoactive drug classes	Proposed mechanism of action (example)
Expectorants	Increase mucus secretion volume and/or hydration for more productive cough (e.g. guaifenesin)
Mucolytics	Reduce mucus viscosity by breaking down tertiary structures within mucus (e.g. N-acetylcysteine)
Mucokinetics	Increase mucus transportability by mucociliary transport and cough mechanisms (e.g. ambroxol)
Mucoregulators	Affect the regulation of mucus synthesis and reduce mucus hypersecretion (e.g. anticholinergic agents)

### Pharmacology

#### Pharmacokinetics

Guaifenesin [3-(2- methoxyphenoxy)-1,2-propanediol] has been well characterized chemically [17]. Animal studies showed that guaifenesin is generally well absorbed and has an established pharmacokinetic profile. In rats, when administered by various routes including intravenous (IV) bolus, oral gavage (50 mg/kg, 25 mg/mL), and gastric, jejunal or cecal infusions (50 mg/kg, 50 mg/mL), guaifenesin achieved a maximum plasma concentration (C_max_) of 15–33 μg/mL [18]. The time to reach C_max_ (T_max_) in rats was faster when given as an oral bolus (27 min) than with gastric, jejunal or cecal infusions (120 min) [18]. In rats, the bioavailability of guaifenesin for all gastrointestinal (GI) routes was ~70%, and the terminal half-life of IV administration (~45 min) was identical to that associated with various GI routes of administration (45–54 min) [18].

Guaifenesin is well absorbed from the human GI tract. Following a single oral dose of guaifenesin in pediatric subjects, C_max_ was reached in approximately 0.5 h and the plasma elimination half-life was approximately 1 h [19]. In adult subjects, C_max_ was achieved in 1.69 h following a single oral dose of IR guaifenesin; the terminal exponential half-life is approximately 0.86 h [20], and the compound is no longer detectable in the blood at 8 h post dose.

Once absorbed, guaifenesin is efficiently metabolized and subsequently excreted in the urine. Guaifenesin is not known to interfere with the cytochrome P450 (CYP) system, nor is it an inhibitor or inducer of this system. Guaifenesin appears to undergo both oxidation and demethylation. The drug is rapidly metabolized in the liver via oxidation to β-(2-methoxyphenoxy)-lactic acid [21]. The demethylation of GGE (hydroxyguaifenesin) is performed by O-demethylase, localized in liver microsomes; approximately 40% of a dose is excreted as this metabolite in the urine within 3 h. O-demethylase seems to be the main enzyme for the metabolism of GGE [22, 23]. Following oral dosing (400 mg), more than 60% of a dose is hydrolyzed within 7 h, with no parent drug detectable in the urine [24]. The major metabolites of guaifenesin (both inactive) are beta-2-methoxyphenoxy-lactic acid [21, 25] and hydroxy-guaifenesin [22].

#### In vitro and animal studies investigating the mechanism of action

To date, several mechanisms of action have been described for guaifenesin. It has been postulated that guaifenesin exerts its expectorant activity via a neurogenic mechanism: a stimulation of vagal afferent nerves in the gastric mucosa activates the gastro-pulmonary reflex, and increases the hydration of airway mucus [26, 27]. In support of this hypothesis, a study in rats demonstrated that oral but not intravenous guaifenesin administration increased respiratory secretions [18].

The viscoelastic behavior of bronchial mucus has important consequences for mucociliary clearance. This mucus is an adhesive, viscoelastic gel, the biophysical properties of which are largely determined by entanglements of long polymeric gel-forming mucins: MUC5AC (expressed in goblet cells) and MUC5B (originating from submucosal glands) [11]. Inflammatory airway diseases and infections cause mucus (including mucin glycoproteins) overproduction and hypersecretion from metaplastic and hyperplastic goblet cells which contributes to mucus obstruction of airways [6]. Medications that decrease viscoelasticity, such as certain mucolytics, may benefit ciliary clearance.

**Fig. 1:**
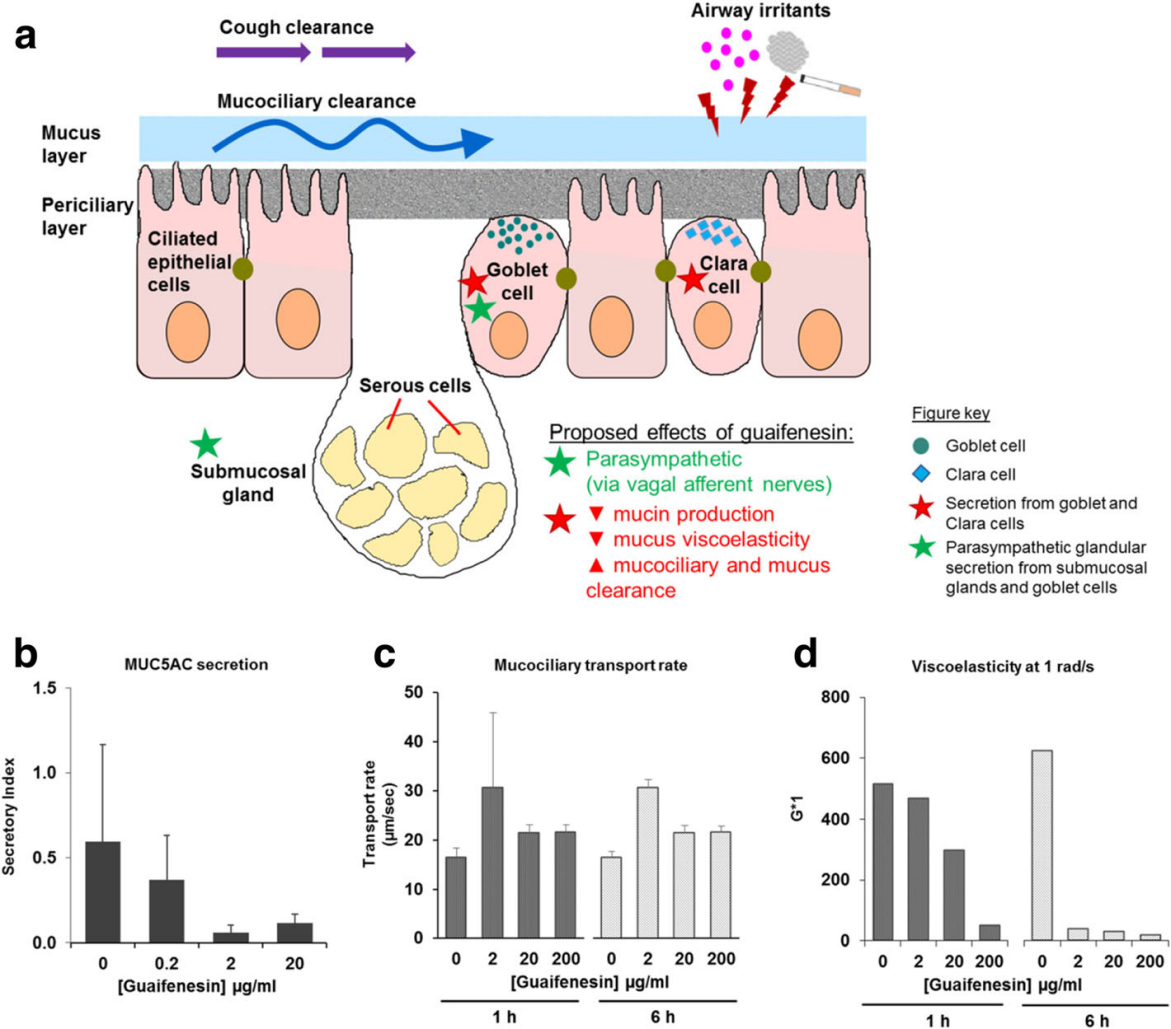
Putative effects of guaifenesin on mucus in chronic or acute hypersecretory respiratory conditions. **a** The airway is composed of a mucus gel layer covering the epithelium, which includes ciliated cells, Clara cells, goblet cells and submucosal glands. The mucociliary complex can be subdivided into two layers – an upper mucus gel layer containing MUC5AC and MUC5B mucins, and a lower layer of periciliary fluid containing cell surface-tethered mucins. Mucociliary clearance (MCC) is effected by the rhythmic sweeping motion of cilia. Prolonged exposure to irritants such as cigarette smoke or allergens can lead to overproduction and hypersecretion of mucus. Guaifenesin has been postulated to promote mucociliary clearance via a number of mechanisms. (1) Indirect activation/stimulation of gastrointestinal vagal afferent nerves triggers reflex parasympathetic glandular secretion from submucosal glands and goblet cells (green stars), increasing hydration of mucus layer for more effective mucociliary clearance. Guaifenesin also affects secretion from goblet and Clara cells (red stars), resulting in (2) decreased mucin production and secretion (green circles, goblet cells; blue squares, Clara cells), and (3) reduced viscoelasticity of mucus, which increases the ability of ciliary movement to remove mucus. Together these changes serve to enhance MCC and mucus clearance. **b**–**d** Guaifenesin has direct effects on MCC-related processes in airway epithelial cells. In cultured human differentiated tracheobronchial epithelial cells, 24-h treatment with guaifenesin (2 or 20 μg/mL) significantly suppressed mucin production and mucin secretion (**b**), while 6-h treatment with guaifenesin (2–200 μg/mL) significantly enhanced mucociliary transport rates (**c**). At 1 h and 6 h after guaifenesin treatment (0–200 μg/mL), significant dose-dependent decreases were observed in mucus viscosity and elasticity at typical ciliary beat frequency (1 rad/s) (**d**), as measured by G*1 (vector sum of viscosity and elasticity at 1 rad/s). Panels b-d adapted from Seagrave et al., 2011 [13]

Recent in vitro studies using differentiated human airway epithelial cells, grown at an air-liquid interface to mimic physiological conditions in the respiratory tract, revealed direct effects of guaifenesin on the airway epithelium [13, 28]. At clinically relevant doses, guaifenesin was found to significantly decrease mucin (MUC5AC) production, mucus viscosity and elasticity, and to enhance MCC [13]. These results were replicated in another study on airway epithelial cells pre-treated with an inflammatory mediator, IL-13, to increase secretions prior to treatment with guaifenesin, N-acetylcysteine, or ambroxol [28]. Guaifenesin was more effective than N-acetylcysteine or ambroxol at increasing MCC rates, inhibiting mucin secretion, and improving mucus rheology. Figure 1 shows some of these putative mechanisms of action (Fig. 1a,-d). Additional in vivo pharmacology and clinical studies will be needed to further elucidate these findings and determine how these mechanisms can be most effectively recruited to produce clinically relevant effects in the target populations.

#### Human studies investigating the mechanism of action of guaifenesin

Studies in patients with chronic bronchitis demonstrated that guaifenesin increases MCC [29] and reduces sputum viscosity [30]. Bennett and coworkers compared the effects of guaifenesin and placebo on in vivo MCC by measuring the rate of removal of inhaled radioactive tracer particles from the lungs of healthy, non-smoking adults. Guaifenesin enhanced small airway clearance with a strong trend toward statistical significance (*p* = 0.07) [31]. In a similar study with a crossover design to assess the effects of guaifenesin on MCC and cough clearance (MCC/CC) in adults with acute RTIs, it was reported that the effect of a single dose of guaifenesin on MCC/CC could not be differentiated from that of placebo in that study population [32].

A study in healthy volunteers with a history of sinus disease did not detect significant differences between guaifenesin and placebo treatment in terms of their effects on in vivo nasal MCC [33]. Saccharin particle transit time (STT) was similar with guaifenesin and placebo, and it was suggested that additional factors could have an impact on MCC and/or ciliary motility.

Guaifenesin has been shown to make coughs more productive [34], and additionally has been found to inhibit cough reflex sensitivity in subjects with acute URTIs [15, 16]. Two double-blind, randomized and placebo-controlled studies investigated the effect of a single dose of guaifenesin (400 mg and 600 mg, respectively) on participants’ response to a nebulized capsaicin cough challenge. Guaifenesin significantly reduced cough reflex sensitivity in patients with viral URTIs [15, 16], but not in healthy volunteers. The authors suggested that this effect was limited to patients with URTIs due to their transiently increased cough receptor sensitivity.

Details of clinical studies mentioned in this section are in Table 3.

**Table 3:** Pharmacology studies

First Author (Year)	Objectives of study	Intervention	Results
Chodosh (1973)	To evaluate sputum changes associated with guaifenesin in chronic bronchial conditions	Double-blind crossover study: 1 week known placebo +800 mg/day or 2400 mg/day for 4 weeks +1 week unknown placebo +1000 mL extra oral water intake	Significantly uniform and beneficial changes, such as sputum adhesiveness and dry weight, occurred with 2400 mg/day guaifenesin
Thomson (1973)	To assess the effect of guaifenesin on mucociliary clearance (MCC) in the human lung from the rate of removal of inhaled radioactive tracer particles	Double-blind crossover study: 600 mg guaifenesin vs placebo	Significant improvement in mucociliary clearance with guaifenesin in patients with chronic bronchitis but not in healthy subjects
Sisson (1995)	To examine: (i) The effect of guaifenesin on nasal MCC in vivo (assessed by saccharin transit time [STT]) and nasal ciliary beat frequency (CBF; assessed by nasal brushing and ex vivo microscopy), and (ii) whether a relationship exists between nasal CBF and nasal STT	Double-blind crossover study: 400 mg guaifenesin vs placebo 5× daily from days 1 to 7, or days 14 to 21	No significant differences between guaifenesin- or placebo-treated groups in change from baseline values of STT or CBF No relationship observed between STT and CBF from regression analysis
Dicpinigaitis (2003)	To evaluate the effect of guaifenesin on cough reflex sensitivity to inhaled capsaicin in healthy subjects and subjects with acute URTIs	Randomized double-blind study: Single dose of 400 mg guaifenesin vs placebo	Cough reflex sensitivity was statistically significantly decreased with guaifenesin in patients with URTIs but not in healthy subjects
Dicpinigaitis (2009)	To evaluate the antitussive effect of the combination of benzonatate and guaifenesin in subjects with acute URTI	Randomized double-blind crossover study (*n* = 23): Each subject received 3 of 4 possible study drug/combinations: 600 mg guaifenesin (G), 200 mg benzonatate (B), their combination (B + G), and placebo (P)	• Guaifenesin (*p* = 0.01) significantly inhibited cough reflex sensitivity relative to placebo • B + G combination suppressed capsaicin-induced cough significantly more than B (*p* < 0.001) or G (*p* = 0.008) alone
Bennett (2010)	To determine whether guaifenesin improves MCC in the healthy lung by assessing the rate of removal of inhaled radioactive tracer particles	Open-label randomized crossover study (*n* = 8): Single dose of 1200 mg guaifenesin vs placebo over 3 weeks; minimum 7-day washout period	Strong trend toward statistical significance (*p* = 0.07) for enhanced small airway clearance with guaifenesin vs placebo
Bennett (2015)	To determine the effect of guaifenesin on MCC and cough clearance in non-smoking adults with acute URTI by assessing the rate of removal of inhaled radioactive tracer particles	Randomized double-blind crossover study: Single dose of 1200 mg guaifenesin vs placebo	No significant effect of single dose guaifenesin on mucociliary and cough clearance compared to placebo

## Clinical efficacy studies in respiratory diseases

### Clinical uses of guaifenesin

Despite the large number of clinical studies on different clinical aspects of guaifenesin therapy, its expectorant indication is currently the only one that the FDA considers to be supported by sufficient medical evidence. The “Cough-Cold” Final OTC Monograph covers the use of guaifenesin in adults and children 2 years and older, and is based on a subset of clinical studies in chronic respiratory diseases that were available when the monograph was developed. The Monograph indication for guaifenesin is limited to symptomatic treatment of acute URTIs and stable chronic bronchitis [3]. The FDA approved labels for guaifenesin include an OTC label for its use in the treatment of chest congestion associated with an URTI but also a professional label for chest congestion associated with stable chronic bronchitis for its detailing to healthcare professionals. This professional label indication mirrors the outcome of clinical studies conducted on chronic bronchitis patients. The exact wording of the indications is listed below:

- Guaifenesin “helps loosen phlegm (mucus) and thin bronchial secretions -
  ○ to rid the bronchial passageways of bothersome mucus and make coughs more productive” (OTC uses)
  ○ in patients with stable chronic bronchitis” (professional indication).

A review of the literature supporting the clinical utility of guaifenesin shows effects across three categories of respiratory conditions: chronic bronchitis and chronic respiratory conditions (Table 4), URTIs (Table 5), and rhinosinusitis (Table 6). Almost all studies discussed here were conducted in adults, with the exception of one published study in children on the use of guaifenesin for relieving cough symptoms [35].

**Table 4:** Clinical efficacy studies: Chronic bronchitis and chronic respiratory conditions

Author (Year)	Objectives of study	Intervention	Results
Hayes (1956)	To determine the effectiveness of Robitussin® as an expectorant in productive cough due to chronic pulmonary disease	1–2 g Robitussin® (containing 100 mg guaifenesin and 1 mg desoxyephedrine HCl per 5 mL) vs placebo every 2–3 h, as required	Statistically significant changes compared to placebo: • Reduction in chronic productive cough • Decreased frequency of cough and sputum viscosity
Chodosh (1964)	To investigate the efficacy and mechanism of action of guaifenesin in bronchopulmonary diseases	Double-blind study: 100 mg guaifenesin tablet 4× daily vs placebo for 14 days (after placebo run-in)	Statistically significant changes compared to placebo: • Increase in ease of expectoration • Decrease in the measured sputum surface tension
Hirsch (1973)	To investigate the expectorant effect of guaifenesin in patients with chronic bronchitis	Single-blind crossover study: 800 mg or 1600 mg guaifenesin vs placebo alternating for 5 weeks Double-blind crossover study: 1600 mg guaifenesin vs placebo daily for 5 days	No significant difference between guaifenesin and placebo in reducing sputum consistency, increasing sputum volume, improving ventilatory function or ease of expectoration
Wojcicki (1975)	To investigate the effect of guaifenesin on: (i) Severity and frequency of cough, and (ii) Tenaciousness of sputum	Double-blind crossover study: 120 mg guaifenesin vs 17 mg narcotine HCl, vs combination (120 mg guaifenesin +17 mg narcotine HCl), vs placebo, 3× daily for 7 days (per treatment)	Guaifenesin + narcotine HCl combination associated with statistically significant decreases in: • Cough severity • Cough frequency
Finiguerra (1982) (unpublished; data on file)	To determine the efficacy of guaifenesin for: (i) Modifying the volume and viscosity of tracheobronchial secretions, and in (ii) Providing symptomatic relief of difficult expectoration and cough in chronic bronchitis	Randomized double-blind parallel-group study: 190 mg guaifenesin vs placebo, 3× daily for 15 days	Statistically significant changes: • Decrease in sputum volume and viscosity • Decrease in cough severity • Improvement in ease of expectoration
Parvez (1996)	To determine the usefulness of a multidimensional cough quantitation system for evaluating guaifenesin’s effects on cough and sputum	Randomized double-blind parallel-group study: 300 mg guaifenesin vs placebo, 4× daily for 14 days	Differences between guaifenesin and placebo groups: • Guaifenesin significantly increased sputum volume; 37% difference on day 14 (*p* < 0.05) • Significant reduction in fucose, a biomarker for sputum glycoprotein, in the guaifenesin group at day 14 (*p* < 0.01) • Subjective measure of average intensity/cough at day 4 (*p* < 0.05) • Trend for greater improvement in ease of expectoration at days 10 and 14 in the guaifenesin group but did not reach significance in the subgroup with productive cough (*p* < 0.01)

**Table 5:** Clinical efficacy studies: Upper respiratory tract infections

First Author (Year)	Objectives of study	Intervention	Results
Robinson (1977)	To confirm that guaifenesin was superior to placebo in facilitating expectoration of sputum and ameliorating dry cough in patients with an acute upper respiratory infection	Randomized double-blind parallel-group study: 200 mg guaifenesin in 10-mL doses vs placebo, 4× daily for 3 days	Subjective measures compared to placebo: • Cough frequency reduced at 48 h, 72 h (all: *p* < 0.01) • Cough intensity reduced at 48 h, 72 h (all: *p* < 0.01) • Chest discomfort reduced at 24 h, 48 h, 72 h (all: *p* < 0.01) • Sputum volume increased (only in patients with productive cough) at 48 h (*p* < 0.01) • Ease of raising sputum increased at 24 h, 48 h, 72 h (all: *p* < 0.01)
Kuhn (1982)	To evaluate the efficacy of guaifenesin in reducing cough frequency in adults with acute respiratory disease	Double-blind study: 2400 mg guaifenesin vs placebo syrup vehicle in 30-mL doses every 6 h for 30 h	• Objective cough counts: No significant differences between guaifenesin and placebo • Significantly greater decrease in sputum viscosity compared to baseline in patients with productive cough (*p* = 0.001) • Greater decrease in sputum quantity (*p* = 0.07)
Albrecht (2012)	Pilot study to determine the efficacy of extended-release (ER) guaifenesin with placebo for treatment of URTI, using objective and subjective efficacy assessments	Randomized double-blind study: 1200 mg ER guaifenesin vs placebo 2× daily for 7 days	Subjective measures of efficacy (patient-reported outcomes; PROs) showed the most prominent differences between treatment groups at Day 4, in favor of guaifenesin. Based on post-hoc analyses focusing on subsets of these PROs, an 8-question PRO tool (SUM8) was validated.

**Table 6:** Clinical efficacy studies: Rhinosinusitis

First Author (Year)	Objectives of study	Intervention	Results
Wawrose (1992)	To evaluate the role of guaifenesin in decreasing symptoms of postnasal drainage and nasal congestion in HIV+ patients with chronic rhinosinusitis	Double-blind parallel-group study: 1200 mg guaifenesin vs placebo 2× daily for 3 weeks	Significantly less nasal congestion and thinner postnasal drainage reported after 3 weeks of treatment with guaifenesin vs placebo (*p* < 0.05)
Rosen (2005)	To evaluate the effect of guaifenesin on mucociliary clearance time (MCT) and sinonasal symptoms in HIV+ patients	Randomized double-blind parallel-group study: 1200 mg guaifenesin vs placebo 2× daily for 3 weeks	Significant improvement in sinonasal symptom survey (SNOT-16) score in HIV+ patients treated with guaifenesin vs placebo (*p* < 0.05)

It should be noted that stable chronic respiratory conditions, such as chronic bronchitis, have proved more reliable as clinical models for studying the effects of expectorants and other mucoactive drugs. Mucus production and associated cough symptoms tend to be more stable in chronic respiratory conditions, allowing the effects of guaifenesin to be observed more consistently.

### Chronic bronchitis and chronic respiratory conditions

The inclusion of guaifenesin in the 1989 Final OTC Monograph was essentially supported by four clinical studies in patients with chronic bronchitis [36–39]. All of these definitive studies demonstrated statistically superior efficacy of guaifenesin versus controls in improving ease of expectoration, decrease in sputum surface tension and viscosity, or reduction in the frequency and severity of cough (Table 4).

An early study in chronic bronchitis patients reported that guaifenesin’s effects on sputum consistency and volume were comparable to that of placebo [40]. Other studies, however, support the drug’s effects on sputum. Although results for cough assessments in patients with chronic bronchopulmonary disease were mixed, guaifenesin-treated patients reported increased sputum volume compared with placebo, as well as greater ease of expectoration [41]. These findings are consistent with an earlier study on objective sputum changes in patients with chronic bronchitis; guaifenesin was found to significantly decrease sputum adhesiveness and quantity (dry weight), and was also reported to improve expectoration [30].

### Acute upper respiratory tract infections (URTIs)

The efficacy of guaifenesin as an expectorant has also been examined in the context of acute URTIs (Table 5). Robinson and coworkers showed that guaifenesin improved acute URTI symptoms based on patient-assessed subjective measures (cough frequency and intensity) and physicians’ evaluation of global effectiveness [42]. In adults with acute URTIs, guaifenesin significantly reduced sputum thickness and quantity compared to placebo [41].

A large placebo-controlled pilot study explored a range of objective and subjective outcome measures in patients with acute URTIs. The most promising measures included a daily diary for patient-reported outcome (PRO) parameters. These described symptoms such as severity of chest congestion, mucus thickness and cough. Some of these 11 exploratory parameters showed strong trends or statistically significant differences between guaifenesin and placebo. A PRO validation process served to qualify more focused subsets of 4 and 8 questions. Based on post-hoc analyses (*p* = 0.038), an 8-question PRO tool (SUM8) was validated and proposed for use in future respiratory studies [43]. To explore effects on sputum as objective endpoints, laboratory analyses were performed on patient mucus samples from the pilot study. The laboratory analyses could not demonstrate differences in mucus properties with guaifenesin compared to placebo; however, it should be noted that methodological issues with mucus sample collection and shipping were present, raising some questions about the interpretation of the laboratory results [44].

### Rhinosinusitis

Guaifenesin was reported to be effective for improving symptomatic rhinitis and sinusitis by decreasing nasal congestion and postnasal discharge in immunocompromised HIV positive patients [45, 46]. Despite some conflicting data available, some patients with rhinitis benefit from using guaifenesin [12]. Further research is needed to clarify guaifenesin’s effects on congestion and mucus clearance from the nasal passages and sinuses in the general patient population.

## Clinical safety

As a single agent, guaifenesin has a well-established and favorable safety and tolerability profile. Its safety record is supported by data from published clinical studies and a history of post-marketing surveillance safety reports covering more than 50 years in the US and around the world. Common side effects reported for the drug include dizziness, headache, and gastrointestinal disturbances at high doses [17].

Retrospective and ongoing prospective pediatric safety data analyses confirm guaifenesin’s favorable safety profile as an OTC drug in children [47, 48]. In a continuous safety surveillance analysis of 8 common cough and cold drugs conducted from 2008 through 2014, guaifenesin showed the lowest number of “at least potentially related” non-fatal adverse event (AE) cases (1%) out of a total of 5610 index drug reports [48]. Guaifenesin had the lowest frequency of mentions for non-fatal AEs by system organ class (SOC) at estimated supra-therapeutic and even at estimated unknown dosing; and the second lowest frequency of mentions for non-fatal AEs by SOC at estimated therapeutic dosing. More importantly, guaifenesin was not mentioned in any “potentially related” fatal cases during the 1991–2008 surveillance period, or during the 2008–2014 detection period.

The few published reports of serious adverse events related to the use of guaifenesin have mostly been in the context of overdose and use as part of multiple-drug combinations for various cough and cold indications. Published reports include renal stone formation with chronic guaifenesin overdose [49], and acute fatal intoxication by a combination of guaifenesin, diphenhydramine, and chlorpheniramine, although the relative contribution of guaifenesin to the fatality could not be determined [50].

Pregnancy category C status for GGE was determined by the FDA based on the absence of definitive studies assessing potential risks to the fetus [3, 51]. Results of a recently published study in female, pregnant rats, after testing very high doses of guaifenesin, suggest that the risk of fetal abnormalities cannot be ruled out [52]. The medical literature and safety databases do not show meaningful signals suggesting a significant risk of fetal development issues after pregnant women used guaifenesin. Thus, caution regarding the use of GGE in pregnant women is warranted [51]. The current labeling (“if pregnant, ask a health professional before use”) is in line with the FDA OTC Monograph and seems to be an appropriate warning telling women to avoid taking the drug during pregnancy.

## Guaifenesin drug products and dosing

Immediate-release (IR) and extended-release (ER) guaifenesin are available in single-agent formulations (Table 7). There are also many popular guaifenesin-containing combination OTC and prescription products available on the market, but these are outside the scope of this article. The dual dosing range of guaifenesin in the US allows patients the flexibility to titrate doses to achieve optimal efficacy. In the US, adults and children above 12 years old may take guaifenesin in oral doses of 200 to 400 mg every 4 h, up to a maximum of 2400 mg over 24 h [17]. Pediatric doses cater to children aged 2–12 years, and differ according to age groups, i.e. 2–6 years and 6–12 years (Table 7). In Canada, guaifenesin is not recommended for children aged 12 years and below. Dosing regimens and daily maximum doses for adults and children above 12 years old in Canada (daily dose of 1600 mg maximum) also differ from those in the US [53].

**Table 7:** Examples of currently available over-the-counter guaifenesin formulations and recommended doses in the US and Canada

Formulation	Population	Recommended doses	Available dosage form – Product example(s)
Immediate-release	Children – 2 to <6 years	US: 50–100 mg up to 4-hourly; max 600 mg/day Canada: Not recommended	Syrup – CVS Health® Children’s Mucus Relief Chest Congestion Soft chews – Kids-EEZE® Chest Relief
Immediate-release	Children – 6 to <12 years	US: 100–200 mg up to 4-hourly; max 1200 mg/day Canada: Not recommended	Syrup – CVS Health® Children’s Mucus Relief Chest Congestion Soft chews – Kids-EEZE® Chest Relief
Immediate-release	Adults and children 12 years and older	US: 200–400 mg up to 4-hourly; max 2400 mg/day Canada: 200–400 mg up to 6-hourly; max 1600 mg/day	Tablet – Bidex® Syrup – Robitussin® Mucus & Chest Congestion; Scot-Tussin Expectorant Soft chews – Kids-EEZE® Chest Relief
Extended-release	Adults and children 12 years and older	US: 600–1200 mg up to 12-hourly; max 2400 mg/day Canada: 600 mg (1 tablet) up to 12-hourly; max 1200 mg/day (2 tablets)	Tablet – Mucinex®

**Fig. 2:**
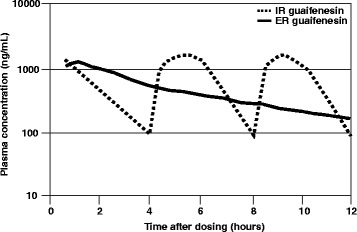
Schematic pharmacokinetic profile of extended-release (ER) vs immediate-release (IR) guaifenesin formulations. Extended-release (ER) guaifenesin (blue line) attained bioequivalent plasma concentrations to those obtained with 3 immediate-release (IR) guaifenesin doses (orange line). The unique bi-layer tablet formulation comprises an IR layer that permits immediate release of guaifenesin to rapidly attain maximum plasma concentrations (C_max_), and an ER layer that permits sustained release of guaifenesin to maintain prolonged blood plasma levels of guaifenesin over 12 h. Figure adapted from Vilson and Owen, 2013 [20]

Because of guaifenesin short half-life, frequent dosing with IR guaifenesin is required to maintain therapeutic levels of the drug in the body (Fig. 2). Subsequently 12-h extended release form of guaifenesin were designed to provide bioequivalent pharmacokinetic characteristics to generic IR guaifenesin products [20] and are currently approved as 12-h tablet ER guaifenesin formulation in the US market. An example of such ER products is a bi-layer tablet formulation containing 600 mg of guaifenesin and comprising an IR layer that allows rapid release of guaifenesin to achieve an early C_max_, and an ER layer that allows sustained release of guaifenesin to produce a steady plasma concentration over a 12-h period (Fig. 2). Following approval of this extended release form of guaifenesin (NDA-21-282) in 2002, the FDA required the removal of all marketed, but unapproved, timed-release guaifenesin products from the market by 2007.

## Conclusions

This review provides an updated and comprehensive perspective on the use of guaifenesin in treating respiratory disorders in which excessive mucus is an important clinical feature. Excessive mucus secretion and local accumulation in the airway occurs in both acute URTIs and chronic respiratory disorders with an underlying inflammatory etiology (such as chronic bronchitis and COPD). The expectorant properties of guaifenesin, which help to thin bronchial secretions and promote mucus clearance, were demonstrated in studies involving patients with chronic bronchitis or other chronic respiratory conditions. These studies played an important role in the FDA’s decision to include guaifenesin as an expectorant in the respective Final OTC Monograph labeled for the relief of mucus-related symptoms of acute URTIs and stable chronic bronchitis. Additional studies have been performed to clarify the mode of action and assess guaifenesin’s efficacy and safety in other clinical indications [13, 28, 29, 42, 45, 46, 54].

Recent advances in the understanding of guaifenesin’s mechanism of action add to the understanding of the drug’s potential in the management of hypersecretory respiratory conditions. Studies in symptomatic chest congestion and acute cough, as well as in acute rhino-sinusitis indications, have yielded mixed results. This may be understandable, given the context of rapidly changing symptoms in acute URTIs, which are challenging to study under standard clinical trial conditions. Some studies showed evidence of efficacy based on improvements in subjective measures as patients assessed their cough, mucus clearance, or chest congestion symptoms. However, in many cases the methods were not validated or results were not confirmed by subsequent studies. For this reason, the effects of guaifenesin have been more consistently demonstrated in stable chronic respiratory disease models. Further research is needed to clarify the antitussive effectiveness of guaifenesin and its ability to relieve chest congestion in acute URTIs in children and adults, and the utility of the drug in improving symptoms of rhino-sinusitis.

To date, the approved indications for guaifenesin have not changed from those included in the 1989 Final Monograph. Interestingly, the secondary indication for stable chronic bronchitis remains largely underutilized or unrecognized even among US medical professionals. Further progress will require improved assessment tools and appropriately designed, modern studies, to confirm guaifenesin’s utility in acute and chronic hypersecretory respiratory conditions.

A large body of AE reporting data supports the safety of guaifenesin for adult and pediatric use. Unlike certain other OTC cough and cold medications, guaifenesin has not been reported to cause many serious side effects or abuse/dependence problems, and has been proven safe in studies for use in conditions such as URTIs and stable chronic bronchitis.

Well-established as a safe expectorant drug, guaifenesin has achieved common usage for the relief of mucus-related symptoms of acute URTIs and for patients with mucus-related symptoms in the context of stable chronic bronchitis. Additional, up-to-date, and high-quality data are needed to explore the full potential of this compound in established uses, and in new respiratory indications associated with mucus hypersecretion.

## Abbreviations

AE: Adverse event
CC: Cough clearance
COPD: Chronic obstructive pulmonary disease
CYP: Cytochrome P450
ER: Extended-release
FDA: Food and Drug Administration
GGE: Glyceryl guaiacolate ether
GI: Gastrointestinal
HIV: Human immunodeficiency virus
IL-13: Interleukin 13
IR: Immediate-release
IV: Intravenous
MCC: Mucociliary clearance
NDA: New drug application
OTC: Over-the-counter
PRO: Patient-reported outcome
STT: Saccharin particle transit time
URTI: Upper respiratory tract infection
